# Caso 5/2020 – Transposição Corrigida das Grandes Artérias em Boa Evolução Natural em Mulher de 65 Anos

**DOI:** 10.36660/abc.20190489

**Published:** 2020-09-11

**Authors:** Edmar Atik, Renato Maluf Auge, Alessandra Costa Barreto, Maria Angélica Binotto

**Affiliations:** 1 Hospital das Clínicas Faculdade de Medicina Universidade de São Paulo São PauloSP Brasil Instituto do Coração do Hospital das Clínicas da Faculdade de Medicina da Universidade de São Paulo,São Paulo, SP – Brasil

**Keywords:** Bloqueio Atrioventricular, Disfunção Ventricular, Transposição Congênita Corrigida de Grandes Artérias/cirurgia, Marca-Passo Artificial, Adulto, Diagnóstico por Imagem

## Dados clínicos

Paciente evolui sem sintomas, nos afazeres habituais como doméstica e costureira, quando foi identificado em avaliação de rotina bloqueio atrioventricular total com frequência cardíaca de 66 bpm. Nesta ocasião foi feito diagnóstico de transposição corrigida das grandes artérias (TCGA) por ecocardiograma, com insuficiência discreta da valva atrioventricular esquerda. Por insuficiente cronotropismo e doença binodal, foi colocado, aos 59 anos de idade, marcapasso atrioventricular à direita. Permanece assintomática em uso de medicação anti-hipertensiva, anlodipina, enalapril e hidroclorotiazida. Nega qualquer sintoma como palpitações, dor precordial ou cansaço.

Exame físico: bom estado geral, eupneica, acianótica, pulsos normais nos 4 membros. Peso: 61 Kg, Alt.: 147 cm, PAMSD: 118 x 70 mmHg, FC: 72 bpm.

Precórdio: *Ictus cordis* na linha hemiclavicular esquerda e algo impulsivo, sem impulsões sistólicas na borda esternal esquerda. Bulhas cardíacas hiperfonéticas, estando a segunda bulha desdobrada. Sopro sistólico +/++/4, suave, mais audível na ponta cardíaca. Cicatriz na região infraclavicular esquerda pela loja do marcapasso. Fígado não palpado e pulmões limpos.

## Exames complementares

**Figura 1:**
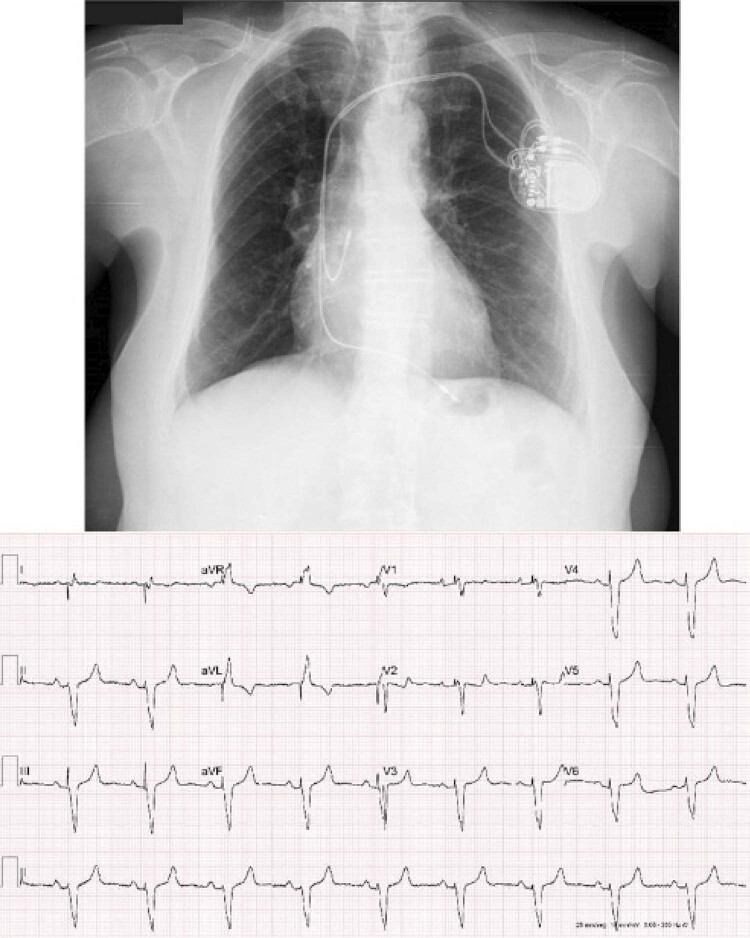
– Radiografia de tórax salienta a área cardíaca discretamente aumentada pelo arco ventricular esquerdo mais saliente e trama vascular pulmonar normal. O arco aórtico se situa à esquerda. Eletrocardiograma demonstra a boa funcionalidade do marcapasso atrioventricular à direita, com bloqueio do ramo direito e posicionado no ventrículo esquerdo à direita.

**Eletrocardiograma:** Ritmo comandado por marcapasso no átrio direito e sinais de bloqueio do ramo direito pela colocação do marcapasso à direita a nível do ventrículo anatomicamente esquerdo ( Figura 1 ).

**Radiografia de tórax:** Aumento discreto a moderado da área cardíaca a custa do arco ventricular esquerdo alongado (ICT=0,68). Trama vascular pulmonar aumentada com arco aórtico à esquerda ( Figura 1 ).

**Figura 2:**
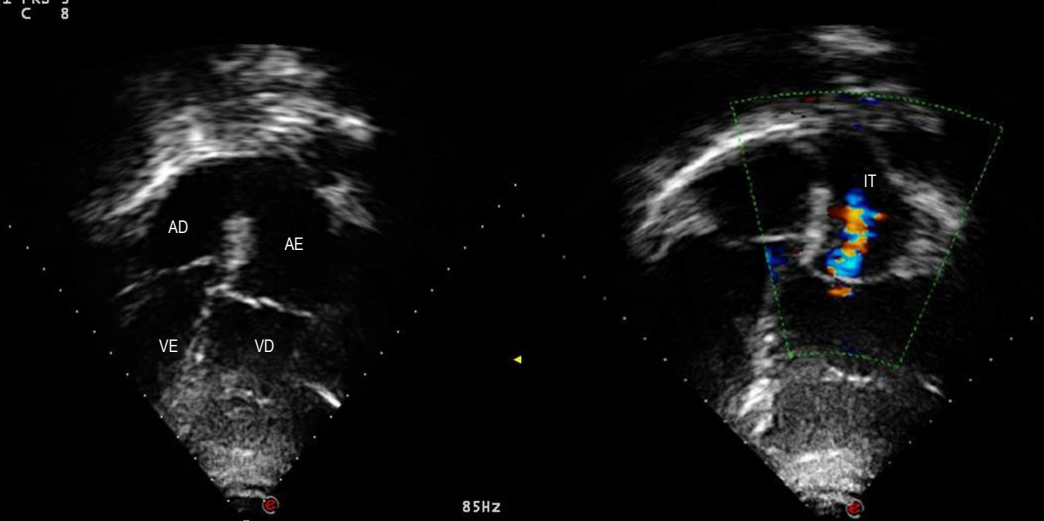
– Ecocardiograma demonstra as imagens características da transposição corrigida das grandes artérias com dilatação discreta do ventrículo direito à esquerda e discreta insuficiência da valva tricúspide à esquerda. A valva mitral à direita é superior e a valva tricúspide mais baixa à esquerda.

**Ecocardiograma** : Conexão atrioventricular e ventrículo-arterial discordantes. Valva tricúspide à esquerda displásica e redundante. Aumento discreto do átrio esquerdo (46 mm com volume= 36 ml/m^2^), e do ventrículo direito à esquerda, estando normais as outras cavidades (VD= 44, VE= 31, Ao= 36) assim como as outras válvulas cardíacas. Não havia hipertrofia miocárdica com septo e parede posterior= 8 mm. A pressão sistólica da artéria pulmonar foi estimada pelo *Doppler* em 26 mmHg. A função biventricular era normal e a fração de ejeção do ventrículo esquerdo de 60%. TAPSE de VD= 1,6 cm e FAC de VD= 40%. ( Figura 2 ).

**Ventriculografia radioisotópica:** Função biventricular normal (VD= 55% e VE= 53%).

**Diagnóstico clínico:** TCGA com insuficiência da valva atrioventricular à esquerda, discreta a moderada intensidade e bloqueio atrioventricular total em evolução natural em paciente de 65 anos, assintomática e com preservação da função miocárdica. Houve colocação de marcapasso atrioventricular à direita aos 59 anos.

**Raciocínio clínico:** Havia elementos clínicos de orientação diagnóstica da cardiopatia congênita, apesar da ausência de sintomas evidentes. A segunda bulha hiperfonética na expressão da transposição das grandes artérias e o sopro sistólico na ponta referente à insuficiência da valva atrioventricular à esquerda seriam os dois elementos diagnósticos da anomalia congênita. Acresce ainda a evolução natural para bloqueio atrioventricular total como elemento também orientador do diagnóstico. Outro elemento seria extraído do eletrocardiograma com potenciais inversos da despolarização e repolarização ventricular, mas não disponíveis. Esse diagnóstico clínico elaborado não pode ser realizado anteriormente à colocação do marcapasso pela falta de sintomas, mas também pela negligência de um exame clínico semiológico devidamente realizado e avaliado com a acurácia adequada. O diagnóstico no caso foi estabelecido pelo ecocardiograma.

**Diagnóstico diferencial:** Outras cardiopatias que se acompanham de hiperfonese de segunda bulha e sopro sistólico sem expressividade poderiam orientar no adulto a cardiopatias que são acompanhadas de hipertensão arterial sistêmica. Dentre as cardiopatias congênitas, lembro as que se operadas conservam a anatomia da transposição arterial como na transposição das grandes artérias submetida à operação de Senning, assim como as operadas sob a técnica cavopulmonar total.

**Conduta:** Em face da boa evolução da paciente na preservação da função normal ventricular direita e sem defeitos cardíacos dignos de significância, a conduta expectante é facilmente adotada com os controles adequados do marcapasso inserido há cerca de 6 anos. Espera-se daí uma continuidade da boa evolução ao longo de muitos anos adiante.

**Comentários:** A TCGA se apresenta de maneira diversa quando se exterioriza com defeitos associados em relação à ausência dos mesmos. Ela simula a tetralogia de Fallot quando a ela se associam a comunicação interventricular (CIV) e a estenose pulmonar, à CIV em presença do mesmo defeito associado e a insuficiência valvar mitral em presença da insuficiência da valva atrioventricular esquerda. Quando a TCGA se mostra sem defeitos associados (15% dos casos) a evolução natural se caracteriza pela evolução do distúrbio da condução atrioventricular, alterado pelo feixe direito muito longo, que favorece o aparecimento do bloqueio atrioventricular total. Ademais, pelo surgimento da insuficiência do ventrículo direito, que pela hipertrofia e dilatação propicia a insuficiência coronária relativa com fibrose e consequente disfunção ventricular. No entanto, raros casos evoluem de maneira mais favorável, como no caso em discussão.

Na literatura, alguns desses casos também tem apresentado tal evolução favorável, citando oito deles recentemente descritos com pouca manifestação clínica^1^ . Além destes, o de maior idade descrito com 83 anos e assintomático^2^ e ainda outro paciente com 70 anos assintomático e com estenose valvar pulmonar associada, com gradiente protetor de 49,9 mmHg entre o ventrículo esquerdo e o tronco pulmonar^3^ . O manejo destes pacientes depende da presença de sintomas, do grau da disfunção ventricular e das intercorrências relacionadas à própria evolução natural do defeito congênito^4^ .
