# Supplementary material for: Caso 5/2020 – Transposição Corrigida das Grandes Artérias em Boa Evolução Natural em Mulher de 65 Anos
Source: Arq Bras Cardiol. 2020 Sep 11;115(1 Suppl 1):34–6. [Article in Portuguese] doi: 10.36660/abc.20190489 (PMC8386953; doi:10.36660/abc.20190489)
Supplement: Translation

# Translation: Case 5/2020 – Corrected Transposition of the Great Arteries, with Good Natural Evolution in a 65-Year-Old Woman

**Authors:** Edmar Atik, Renato Maluf Auge, Alessandra Costa Barreto, Maria Angélica Binotto

**Affiliations:** 1 Brazil Instituto do Coração do Hospital das Clínicas da Faculdade de Medicina da Universidade de São Paulo, São Paulo, SP – Brazil

**Keywords:** Atrioventricular Block, Ventricular Dysfunction, Congenitally Corrected Transposition of the Great Arteries/surgery, Pacemaker Artificial, Adult, Diagnostic Imaging

## Clinical data

The patient evolved without symptoms, doing her usual activities of daily living, such as housekeeping and dressmaking, when total atrioventricular block was identified during a routine evaluation with a heart rate of 66 bpm. At this time, a diagnosis of corrected transposition of the great arteries was made through the echocardiogram, with mild left atrioventricular valve regurgitation. Due to insufficient chronotropism and binodal disease, an atrioventricular pacemaker was placed on the right when she was 59 years old. She remained asymptomatic using antihypertensive medication, amlodipine, enalapril and hydrochlorothiazide. She denied any symptoms such as palpitations, chest pain or fatigue.

Physical examination: good overall status, eupneic, acyanotic, normal pulses in the four limbs. Weight: 61 Kgs, Height: 147 cm, BP=118x70 mmHg: 118 x 70 mm Hg, HR: 72 bpm.

Precordium: apical impulse on the left hemiclavicular line and somewhat impulsive, without systolic impulses on the left sternal border. Hyperphonetic heart sounds, with splitting of the second sound. Mild systolic murmur +/++/4, more audible at the cardiac apex. Scar in the left infraclavicular region by the pacemaker implantation. Nonpalpable liver and clear lungs.

## Complementary examinations

**Electrocardiogram:** Cardiac rhythm controlled by a pacemaker in the right atrium and signs of right bundle-branch block due to pacemaker implantation on the right at the level of the anatomically left ventricle ( Figure 1 ).

**Figure 1:** – Chest radiography highlights the slightly enlarged cardiac area due to a more prominent left ventricular arch and normal pulmonary vascular network. The aortic arch is located on the left. The electrocardiogram shows the good right atrioventricular pacemaker functionality, with right bundle-branch block and positioned in the left ventricle on the right.

**Chest X-ray:** Mild to moderate increase in the cardiac area due to an elongated left ventricular arch (CTI = 0.68). Increased pulmonary vascular network with aortic arch on the left ( Figure 1 ).

**Echocardiogram** : Discordant atrioventricular and ventriculoarterial connections. Dysplastic and redundant tricuspid valve on the left. Slight enlargement of the left atrium (46 mm with volume = 36 mL/m^2^), and of the right ventricle on the left, whereas the other cavities were normal (RV=44, LV=31, Ao=36 mm), as well as the other heart valves. There was no myocardial hypertrophy with septum and posterior wall = 8 mm. The pulmonary artery systolic pressure was estimated by Doppler at 26 mm Hg. Biventricular function was normal, and the left ventricular ejection fraction was 60%. RV TAPSE = 1.6 cm and RV FAC = 40%. ( figure 2 ).

**Figure 2:** – Echocardiogram shows the characteristic images of corrected transposition of the great arteries with slight right ventricle dilation on the left and mild tricuspid valve regurgitation on the left. The mitral valve on the right is upper and the tricuspid valve is lower, on the left.

**Radioisotope ventriculography:** normal biventricular function (RV = 55% and LV = 53%).

**Clinical Diagnosis:** Corrected transposition of the great arteries with left atrioventricular valve regurgitation, of mild to moderate intensity, and total atrioventricular block showing natural evolution in an asymptomatic 65-year-old patient, with myocardial function preservation. An atrioventricular pacemaker was implanted on the right at 59 years of age.

**Clinical Reasoning:** There were clinical elements that led to a diagnosis of congenital heart disease, despite the absence of evident symptoms. The second hyperphonetic sound in the expression of the transposition of the great arteries and the systolic murmur at the apex related to the atrioventricular valve regurgitation on the left would be the two diagnostic elements of the congenital anomaly. Moreover, there was a natural evolution to total atrioventricular block during patient evolution as an element that also led to the diagnosis. Another element would be extracted from the ECG with inverse potentials of ventricular depolarization and repolarization, but these are not available. This intricate clinical diagnosis could be performed prior to the pacemaker placement due to the lack of symptoms, but also due to the lack of a semiological clinical examination adequately performed and evaluated with adequate accuracy. The diagnosis in this case was established by the echocardiogram.

**Differential diagnosis:** Other cardiopathies that are accompanied by a hyperphonetic second sound and systolic murmur without significance could lead to heart disease in adults that are accompanied by systemic arterial hypertension. Among the cardiopathies, one might recall the ones that, when operated, preserve the anatomy of arterial transposition, as in the transposition of the great arteries submitted to Senning’s operation, as well as those operated using the total cavopulmonary technique.

**Conduct:** Considering the patient’s good evolution in the preservation of good right ventricular function and with no significant heart defects, the expectant conduct is easily adopted, with the adequate controls of the pacemaker implanted approximately 6 years ago. Hence, the good evolution is expected to continue for many years to come.

**Comments:** The corrected transposition of the great arteries (CTGA) presents itself in a different way when externalized with associated defects in relation to their absence. It simulates Tetralogy of Fallot when it is associated with interventricular septal defect and pulmonary stenosis, VSD in the presence of the same associated defect and mitral valve regurgitation in the presence of left atrioventricular valve regurgitation. When CTGA shows no associated defects (15% of cases), the natural evolution is characterized by the evolution of the atrioventricular conduction disorder, altered by the very long right bundle, which favors the appearance of total atrioventricular block. Moreover, due to the emergence of the right ventricular insufficiency, which, due to hypertrophy and dilation, leads to relative coronary insufficiency with fibrosis and consequent ventricular dysfunction. However, rare cases have a more favorable evolution, as in the case discussed here.

In the literature, some of these cases have also shown such a favorable evolution, citing eight of them recently described with little clinical manifestation^1^. In addition to these, the oldest one was described at 83 years old and asymptomatic^2^and yet another patient at 70 years old, asymptomatic and with associated pulmonary valve stenosis, with a protective gradient of 49.9 mmHg between the left ventricle and the pulmonary trunk^3^. The management of these patients depends on the presence of symptoms, the degree of ventricular dysfunction and the complications related to the natural evolution of the congenital defect^4^.
